# Evo Devo of the Vertebrates Integument

**DOI:** 10.3390/jdb11020025

**Published:** 2023-06-05

**Authors:** Danielle Dhouailly

**Affiliations:** Department of Biology and Chemistry, Institute for Advanced Biosciences, University Grenoble-Alpes, 38700 La Tronche, France; danielle.dhouailly@univ-grenoble-alpes.fr

**Keywords:** cornea, development, evolution, feather, hair, placode, reticula, scale, tooth

## Abstract

All living jawed vertebrates possess teeth or did so ancestrally. Integumental surface also includes the cornea. Conversely, no other anatomical feature differentiates the clades so readily as skin appendages do, multicellular glands in amphibians, hair follicle/gland complexes in mammals, feathers in birds, and the different types of scales. Tooth-like scales are characteristic of chondrichthyans, while mineralized dermal scales are characteristic of bony fishes. Corneous epidermal scales might have appeared twice, in squamates, and on feet in avian lineages, but posteriorly to feathers. In contrast to the other skin appendages, the origin of multicellular glands of amphibians has never been addressed. In the seventies, pioneering dermal–epidermal recombination between chick, mouse and lizard embryos showed that: (1) the clade type of the appendage is determined by the epidermis; (2) their morphogenesis requires two groups of dermal messages, first for primordia formation, second for appendage final architecture; (3) the early messages were conserved during amniotes evolution. Molecular biology studies that have identified the involved pathways, extending those data to teeth and dermal scales, suggest that the different vertebrate skin appendages evolved in parallel from a shared placode/dermal cells unit, present in a common toothed ancestor, c.a. 420 mya.

## 1. Introduction

The vertebrate integument forms the external body envelope, which creates the boundary between the organism and its environment. It includes both the epithelium, derived from the ectoderm, and the underlying mesenchyme, derived from diverse sources, depending on the anatomic region. The most extensive anatomic constituent is the skin, including both the epidermis, the dermis, and its appendages: glands, scales, feathers, or hair follicle/gland complexes. These structures facilitate a broad range of functions, such as protection, thermoregulation, communication, and locomotion. Integumental surfaces also include a transparent part (the cornea) as well as the anterior three-quarters of the oral cavity, comprising the gingiva and its appendages (the teeth). As a skin embryologist, throughout my scientific life, I have been fascinated by the problem of the vertebrate integument regionalization and its paleontological origin.

Understanding the evolution of the integument is complicated not only by the limited number of species studied in embryology, but also by the limited fossil records. Gland impressions are rare and even controversial, corneous appendages seldom fossilized, and even the homology of mineralized scales can be uncertain. The amphibian skin is defined by its glabrous and glandular nature. While the study of amphibian eggs development laid the foundation of embryology more than a century back, amphibian skin has only been instrumental to morphogenetic analysis [1,2]. However, during metamorphosis, the passage from unicellular to multicellular glands could help suggest similarities to what happened at the passage to terrestrial life. Osteoderms, literally “bones in dermis”, are present in extinct and extant different clades, but are non-homologous structures that might have independently evolved several times. The knowledge of osteoderm morphogenesis in extant species is very limited. At least some osteoderms derive from cartilaginous precursors, as in turtles [3], but others, such as alligator osteoderms, appear to arise via dermal cell condensation [4]. In birds, a great diversity of cutaneous appendages occurs. Indeed, they are not only characterized by feathers, plantar reticula, and beaks, but also have claws, different types of combs and wattles, and according to the species, shanks covered (or not) with overlapping scales [5]. Likewise, mammals possess not only hair and mammary glands, but also show a polymorphism in their corneous appendages [6,7,8,9], which is ancient [10]. This polymorphism involves hair, nails, claws, hoofs, spines, horns, scale-like structures, and the baleen plates of whales. 

An evolutionary theory should be anchored in phylogenetic history. Basal amniotes split into synapsids, which gave rise to mammals, and sauropsids. Very rapidly after the first sauropsids, they split into squamates and archosaurs. Subsequently, archosaurs generated crocodilians and ornithodires, with the latter giving rise to pterosaurs and dinosaurs, including birds. While squamates and archosaurs are diapsids, the chelonian are anapsids, and their relationships concerning the other sauropsids are still debated. Keratins, composed of alpha-polypeptides (“alpha-keratins”) are an evolutionary novelty of vertebrates and characterize all the epithelial cells [11], which are named keratinocytes in the epidermis. The terminal differentiation of keratinocytes is different in anamniotes versus amniotes: the water to land transition was shown to go with a progressive increase in the types and number of proteins that interact with the intermediate filaments of keratins, transforming the process of keratinization into cornification [12,13]. Moreover, sauropsids’ and synapsids’ lineages differ in the composition of their epidermal differentiation complex (EDC) responsible for cornification. This complex involves KAPs (high sulfur, ultra-high sulfur, and high glycine-tyrosine-rich proteins) in synapsids [14], while beta-plated-proteins (formerly “beta-keratins”) are an evolution novelty of sauropsids [15,16]. Epidermal scales, feathers, and hairs are cornified structures, and did not fossilize as well as mineralized teeth, which is even true for the fine sandstones of the Liaoning province of China. Synapsids and sauropsids also differ by the distribution of their cutaneous glands, which are rare in sauropsids but appear in large numbers and diversity in synapsids. Besides mammary, apocrine, and eccrine glands, sebaceous glands are required for the working mechanism of the hair follicle. Indeed, all mouse lines with sebaceous gland deficiency [17,18] present with progressive scaring alopecia, as the release of the hair shaft depends on selective digestion of the inner root sheath by sebum proteases.

This review is based on developmental results, which, with the except of zebrafish, concern the amniotes: lizard, chick, and mouse embryos, a few natural mutations in their corresponding clades, and on fossils of their lineages ancestors, to gain understanding of the evolutionary relationships between different kinds of integument in vertebrates. I fundamentally propose that the squamate epidermis is primarily programmed to form epidermal scales, the avian epidermis to form feathers, and mammalian epidermis to form hairs. The formation of other ectoderm derivatives, such as cornea, mammary gland, and plantar/palmar skin, is driven by negative regulatory mechanisms modulating this default program. However, the relationship between these default potentialities of the epidermis and the odontogenic potential of the oral epithelium is still a mystery.

## 2. Specification of Special Area of the Integument Largely Precedes the Formation of Clade Typical Appendages

Skin is the last organ to differentiate during vertebrate development. In zebrafish, scale formation is even initiated at post-embryonic stages, in 8 to 8.5 mm length larva [19,20,21], while tooth initiation occurs in the 80 h post-fertilization of the embryo [22]. In *Lacerta muralis*, scale formation starts at 20 days post-egg laying, at 26 °C, while the atypical skin areas of the integument are already differentiated [23]. Such a discrepancy in timing between those area and typical cutaneous appendages morphogenesis has been well-documented in chicks and mice. Morphogenesis of feathers begins at E7 for chicks. Vibrissae began at E12.5 and pelage hair began at E14.5 for mice. The specification of corneal ectodermal cells was shown to occur in chick embryos as soon as the neurula stage and the cornea were finally committed by E4–E5. The upper and lower beak form at E4/E5 in chick embryos, following the fusion of facial primordia [24]. In mouse, tooth, and mammary glands, ectoderms are determined at E10. In both species, the fate of the plantar skin appears to be determined with the limb bud formation and orientation at E4.5 in chicks and E10.5 in mice. Thus, all the non-typical skin areas of avian and mammalian integuments are specified before the appearance of feather or hair primordia. How is the identity of these special ectoderm areas acquired on a genetic basis and when was this specialization acquired during evolution?

### 2.1. Corneal Ectoderm

As long as 580 mya, at the stage when the deuterostomes split from protostomes, the common ancestors of those primitive organisms might have already possessed photoreceptors and an associated transparency of their covering ectoderm, like modern simple eyes, as in Medusa. The major discovery of Walter Gehring [25] showed that flies and vertebrates share a gene called *Pax6,* which is involved in eye development. All modern-jawed vertebrates possess sophisticated eyes like the mammalian eye, which suggests that their common ancestor around 420 mya was equipped with such a structure. Thus, the acquisition of the cornea identity by the ectoderm must be very ancient and common, at least to all the vertebrates. Corneal epithelium is first defined by the expression of *Pax6,* and when differentiated, by a pair of keratins, K12/K3, common to all the vertebrates [26]. The expression of *Pax6* occurs very early in corneal ectoderm. This was discovered thanks to the facility of experimentation with first stages in chick embryo development (for a review, see [27]). In 18 h, at the neurula stage, a pre-placodal domain forms next to the neural crest and further splits into the five different sensory placodes [28]. As soon as E2, cells expressing *Dlx5* and *Pax6* segregate to give nasal and “lens” ectoderm [29], respectively, the latter turned out to be corneal ectoderm. According to an old dogma the cornea forms secondarily, induced by the lens. This idea was shown to be wrong. In fact, it is exactly the opposite: the chick corneal ectoderm, expressing *Pax6*, will give rise to the lens at E3, under a BMP4 signal from the optic vesicle [30]. Finally, neural crest cells migration forms the corneal stroma, which inhibits miR-450b-5P, allowing for Pax6 stabilization and the establishment of corneal identity [31]. 

Birds and mammals present different potentials of the corneal epithelium before and after its stroma formation. Early at E3 in chick embryos and E12 in rabbit embryos, the prospective corneal epithelium associated with a E7 chick or E14.5 mouse dorsal dermis rapidly loses *Pax6* expression and is transformed into an epidermis, certified by *keratin 10* (K10) expression, and feathers or hair follicles/sebaceous glands differentiation [30,32,33]. During the fifth day of incubation, i.e., during stroma formation, the chick corneal epithelium loses the capacity to down-regulate *Pax6* and becomes committed. In contrast, rabbit corneal epithelium from an E17–E20 embryo, or even from an adult, is still able to give rise to a hair-bearing skin [32,33]. Indeed, all the basal cells of the adult rabbit central corneal epithelium undergo a multistep process of dedifferentiation under the control of Wnt signals from the associated embryonic mouse dorsal dermis. Shortly after recombination, there was a large increase in the levels of cytoplasmic β-catenin in all the corneal epithelium basal cells. As soon as 2 days after recombination, *Pax6* expression was downregulated and re-localized in the cytoplasm in all the basal cells. From 8 to 12 days the epithelium begins to form hair pegs [33]. Thus, the formation of hair follicles by the adult central corneal epithelium does not appear due to the activation of hypothetical sparsely distributed stem cells in the central cornea, as was postulated by another group [34].

**Figure 1:**
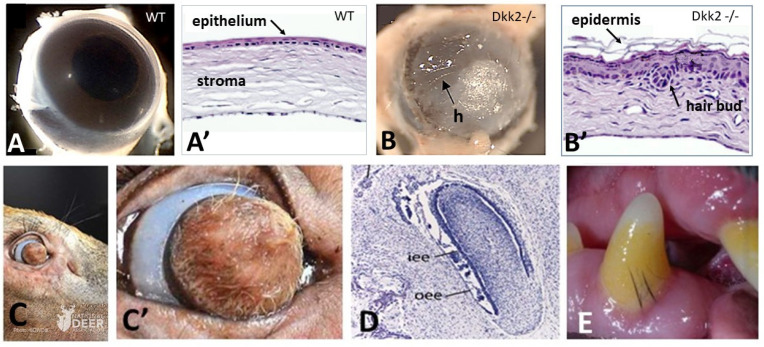
In mammals, a balance between hair and cornea, as well as between hair and teeth. (**A**,**A’**,**B**,**B’**) In mutant mice, when *Dkk2*, a *Wnt* signaling inhibitor, is ablated, the cornea is transformed into a hairy skin. h: hair. (**C**,**C’**) A similar phenotype was observed in a wild deer with hair covering its cornea. (**D**) A transient tooth bud in a bowhead whale fetus: iee: inner enamel epithelium, oee: outer enamel epithelium. (**E**) Subgingival hair associated with a canine in a Labrador retriever dog. **Credits:** (**A**,**A’**,**B**,**B’**): After Mukhopadhyay et al. 2006, [35], reprinted with permission of the Company of Biologists. (**C**,**C’**): By courtesy of Lindsay Thomas, Chief Communications of the National Deer Association (USA). (**D**) After Thewissen et al. 2017 [36], reprinted with permission of John Wiley and sons. (**E**) By courtesy of Jan Bellows, medical director at All Pets Dental in Weston, Florida (USA).

In *Dkk2* knockout mice embryos, a complete transformation of the cornea into skin with developed hair follicles was observed (Figure 1A,A’,B,B’) [35]. It is well-known that the Dickkopf family regulates Wnt pathways by interacting with the Wnt co-receptor LRP5/6. Therefore, one of the requirements for cornea morphogenesis is the expression of *Dkk2* to counteracting *Wnt* expression, to block the β-catenin pathway, fundamentally required for cutaneous appendages formation. A deer with a hair follicle bearing skin instead of cornea (Figure 1C,C’) was recently found (2021, National Deer Association). It could be assumed that natural mutation might have prevented the expression of *Pax6* in corneal ectoderm by enhancing the Wnt/β catenin pathway in the corneal stroma.

### 2.2. Oral Ectoderm

All living jawed vertebrates (gnathostomes) possess teeth or did so ancestrally (birds, turtles, whales). Teeth appeared in the common ancestor of gnathostomes, around 420 mya. The evolutionary origin of teeth has been beset by controversy with competing hypotheses advocating their origin as external tooth-like denticles (“outside-in”) versus de novo independent origin, i.e., from gill arch structures (“inside-out”) [37,38]. This controversy is apparently a false one [39]. Skin denticles can be defined as structures first developing inside and out, united by sets of co-expressing genes, defining a competent epithelial placode and a collaborative set of mesenchymal cells. Current sharks and rays have not only oral teeth but are the only extant lineage, having conserved skin denticles—having been termed placoid scales and called now odontodes—all over their body. 

Tooth development has been extensively studied in mouse embryos, especially by the Irma Thesleff laboratory (for a review: [40]). At E11, the ectoderm gives rise to dental lamina, a stripe of stratified epithelium along the mandible and maxilla, which was later fractioned in tooth placodes. Teeth thus form as appendages of the embryonic oral ectoderm, and their early morphogenesis consists of placodes and typical gene expression like those of other ectodermal appendages [41,42]. Dental lamina was defined one day before its stratification, at E10, as shown by the expression of *Pitx2*. This transcription factor is the most specific marker of epithelium at different stages of tooth morphogenesis, not only prior to dental lamina formation but until amelogenesis. It should be noted that the epithelium is responsible for tooth initiation, as a non-dental mouse mesenchyme can respond to odontogenic epithelial signals [41]. However, the genetic basis of the initial epithelial odontogenic potential of the oral ectoderm, before switching to the mesenchyme, is still unknown, thus precluding experimental mutations studies.

As chondrichthyans ancestors have their entire body covered with odontodes, a first hypothesis is that the odontogenic potential of the body ectoderm was restricted to the oral epithelium in the common ancestor of actinopterygians and tetrapods by 375 mya. The loss of odontogenic potential by the body ectoderm might have led in actinopterygians to the acquisition of potential elasmoid scales, and in the different tetrapods lineages to the acquisition of multicellular glands, epidermal scales, feathers, or hair potentials, respectively. Another explanation could be that the morphogenesis of the body clade specific appendage is prevented in oral epithelium by a still-unknown mechanism. The morphological gap between tooth and hair follicle appears unbridgeable, but in a single case hair type structures were observed to succeed to teeth during development, and in a few cases these two structures were shown to coexist. In the upper jaw of developing extant whales, baleen plates composed of alpha-keratin filaments [43] replace several dozens of transient embryonic tooth buds (Figure 1D), a process dating back to 34 mya [36,44]. Moreover, subgingival typical but short hair associated with teeth have been observed sporadically in modern species, including humans [45,46], old mice (personal communication, Laurent Viriot, ibcp, Lyon, France), and Labrador dogs (personal communication, Jan Bellows, All Pets Dental in Weston, Florida) (Figure 1E). Both observations are in favor of a mechanism preventing hair formation in the oral epithelium. Simultaneous differentiation of both hair and teeth could be explained by a possible sporadic failure of that mechanism. Likewise, the succession of teeth and cornified appendages, only observed in modern whales, is in favor of an exceptional compromise of the mechanisms preventing hair formation in the oral epithelium during post-embryonic development. 

### 2.3. Avian Beak

Avian beaks have many functions, including preening, fighting, and courtship, in addition to feeding, and has permitted birds to diversify into a range of disparate ecological niches. A beak involves three major components—the skeleton of upper and lower mandibles, the epithelium inside the oral cavity, and the horny sheath made of corneous beta proteins—the latter resulting from proliferation of the epidermal cells and cornification of their upper strata. Morphogenesis of the beak results from early signaling interactions among the forebrain, the neural crest cells, and the adjacent surface ectoderm [24]. In chickens, the beak is well-individualized, and its growth starts by the end of embryonic day 5 (E5). It is well-known that birds lost their teeth around 100 mya. The evolution from snout to beak appeared independently, in parallel to the different clades of dinosaurs. It comprises the formation of the horny sheath and the progressive loss of teeth, first restricted to the distal end of the beak [47]. However, a latent property toward tooth formation could remain in oral epithelium of extant birds. Experiments of mouse–chick chimera with early embryos have shown that the avian oral ectoderm kept its odontogenic potential, while the neural crest derived avian mesenchyme lost its capacity to respond to this signaling [48].

### 2.4. Mammary Ectoderm

Another early definition of a particular ectodermal field occurs in mammals: the mammary lines, giving rise to the mammary glands. In mouse embryos, beginning about the time of limb bud formation, at E10.5, the two mammary lines express *Wnt10b* and are specified in part by Fgf10 signaling from the lateral part of the somite [49]. The absence of Shh signaling may be a condition that allows a mammary vs. hair placode fate. Once formed, the mammary bud produces PTHrP, which interacts with BMP signaling to suppress hair formation in the vicinity of the mammary sprout [50]. 

### 2.5. Palmar/Plantar Epidermis

**Figure 2:**
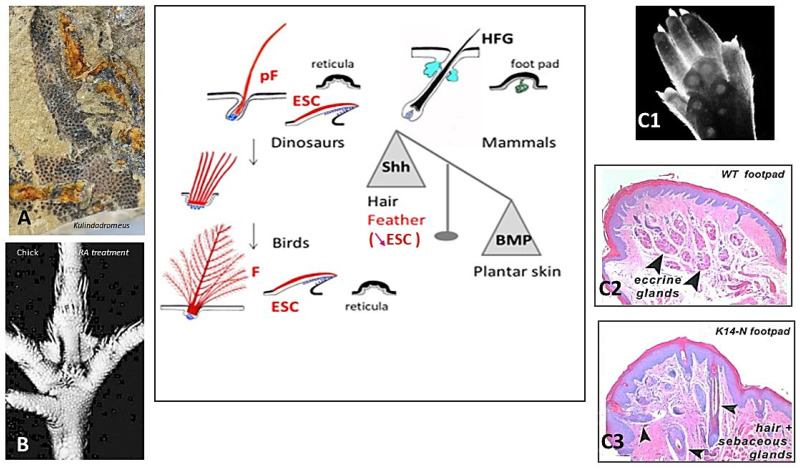
Terrestrial life has been promoted by the development of plantar domains, regulated by the same Shh/BMP balance, both in bird lineage and mammals. (**A**) Basal ornithischian dinosaur *Kulindadromeus* shows plantar reticula. (**B**) In chick embryos, the treatment by retinoic acid at E11 enhances the amount of Shh in skin and leads at E17 to the formation of feathers instead of numerous reticula. (**C1**–**C3**) In mice, the overexpression of Noggin under K14 promoter inhibits BMP4, leading to the formation of hair follicles in the foot pads. Schema: It should be noted that the amount of *Shh* expression in overlapping scale of birds is intermediate between feather (high) and reticula (low). ESC: epidermal scale, HFG: hair follicle gland complex, PF: protofeather, F: feather. Credits: (**A**) by courtesy of P. Godefroit, (**B**) image Dhouailly, (**C1–C3**) After Plikus et al., 2004, [57], reprinted with permission from Elsevier. Schema: Dhouailly.

The avian distribution of feathers appears to be prevented at various degrees on their feet, leading to overlapping scales on shank, and completely on their plantar surface, leading to small cornified bumps, named reticula, which provide cushioning and grip during locomotion and perching. All birds possess reticula on their plantar surface, while several species, such as owls, do not harbor pedal scales. Typical reticula were already present in the ventral surface of the paw of Kulindadromeus (Figure 2A), a basal ornithischian, far from the saurischian lineage leading to birds [51]. Often qualified as scales, the reticula do not contain beta-proteins (among others: [52]). Overlapping scales primordia appear on the chick shank of non-ptilopody breads at E9, and the first reticula at E11 in the central foot pad [53]. However, the fate of the hindlimb integument is linked to the formation and orientation of the limb bud and was thus determined as early as E4.5. It is well-known that En-1 is expressed at E4.5 throughout the chick ventral limb with bud ectoderm. This expression disappears later during chick limb outgrowth and then reappears uniformly at E10 in the ventral foot epidermis, concomitant with the formation of plantar foot pads [54]. The factor linked to En1 reactivation remains to be determined. This strong expression becomes subsequently punctuated signaling the individualization of reticula placodes (see Section 5), which are not histologically distinct, between E10 and E14 [54,55]. In E2 chick embryo, mouse *En1*-transfection on dorsal hind limb presumptive area leads to the downregulation of *Shh* expression and to reticula formation on the dorsal surface of the foot [54]. Reversely, the enhancement of *Shh* expression in plantar skin by retinoic acid treatment at E11/E12, when reticula are about to form, transforms them into feathers (Figure 2B) [53,55]. When a E11 chick plantar dermis is isolated from its epidermis, and was then associated with an umbilical cord epithelium, feathers formed [55]. Thus, feather formation is arrested in the plantar region by an epidermal mechanism preventing a high *Shh* expression, and finally outgrowth. Therefore, I proposed a long time ago that reticula are feathers arrested by an epidermal mechanism, which at least involves the placodal expression of *En-1* [56]. 

The role of En-1 signaling in dorso-ventral limb bud patterning is also well-established in mice. The loss of Engrailed-1 functions results in dorsal transformations of ventral paw structures [58]. Most mammals, like mice, have glabrous foot pads with well-developed sweat glands (Figure 2C1,C2), while a smaller collection, including rabbits, have plantar hairy skin. A sweat gland–hair metaplasia occurs when *Noggin*, a BMP antagonist, is over-expressed in the plantar epidermis (Figure 2C3) [57]. Using inducible and tissue-specific transgenic and lentivirus technology to perturb morphogen signaling levels at different times and in different locations, Lu et al. have shown in detail how BMP works with En1, WNT, FGF, and Shh in a circuit leading to plantar sweat gland formation [59]. This balance between Shh and BMP signaling, at the basis of hair follicles or sweat glands morphogenesis, is also operational to choose between feathers and reticula formation. The acquisition of terrestrial life might have led about 314 mya in basal amniotes, the common ancestor of synapsids and sauropsids, to the prevention of long cutaneous appendages in palmar/plantar skin, such that both modern birds and mammals share the involvement of the same inhibitory mechanisms. However, it is possible that this is only a convergence, as in another sauropsid lineage, which lead to modern lizards, the adaptation to locomotion of the palmar/plantar surface appears totally different. In geckos, the adhesion setae of the digital pads result from an extraordinary transformation of the external cornified layer of the scales, forming numerous long cornified outgrowths over the scale surface [60]. Their timing of determination and their genomic support remain unknown. 

## 3. Skin Appendages: Different Scales, Feathers, and Hair

All skin appendages formation results from a set of successive dermal/epidermal interactions, based on the same pathways and initiated at a placodal stage—see Section 4 and Section 5. The main difference between actinopterygians and amniotes is the last responding tissue of these interactions: the dermis for the mineralized fish scales and the epidermis for the amniote cornified appendages, epidermal scales, feathers, and hair. Among the different types of vertebrate skin appendages, one is special, both for its composition and its paleontological significance—the odontodes.

### 3.1. Fish Skin Appendages: Odontodes and Elasmoid Scales

Like amniote skin appendages, elasmoid scales of actinopterygians [19] and odontodes of chondrichthyans [61] develop relatively late in ontogeny and are distributed across the skin in a hexagonal pattern. The odontodes are dentine and enamel-rich tooth-like structures which evolved over 420 mya. Sharks and rays have retained these ancient skin appendages which are not only tooth-like in structure but share an ancient developmental gene set that is likely common to all cutaneous appendages [61]. One of the earliest bony fishes that lived more than 400 mya, *Lophosteus*, possesses both teeth and odontodes. Its analysis showed that teeth and odontodes initially take shape together but differentiate as they grow [62]. The results confirm that contrary to the ‘scale-to-teeth’ hypothesis, teeth did not evolve from fully formed odontodes. The two structures form out of one founder: teeth and odontodes are modifications of a single system.

Sire and Huysseune [63] have proposed a scenario for the evolution of elasmoid scales, from the superficial odontodal tissue covering the rhombic scales in ancestral osteichthyans. They suggest that teleosteans elasmoid scale tissues are derived from dental but not from bony tissues. In most species, the elasmoid scale is composed of three tissues, the basal plate, composed of elasmodin, the external layer, calcified extracellular matrix like the dentin, and the limiting layer, a hyper-mineralized tissue devoid of collagen fibrils, structurally close to enamel, and deposited on the scale surface by the epidermis. The elasmoid scale is in the upper region of the dermis, close to the epidermis, which still covers most part of its surface in the adult. In the embryo, the epidermis cooperates with the dermis for its morphogenesis. The genes that control the first stages of the fish scales and the appendages of amniotes are the same, as beautifully shown first by Harris and colleagues for ectodysplasin [64], and they interact in similar ways, which have been elegantly demonstrated recently [21] (see Section 5).

### 3.2. Amniotes Scales, Feathers, and Hair

A long-held view is that feathers and hairs have been suggested to evolve from epidermal overlapping scales of a common tetrapod ancestor of sauropsids and synapsids [65]. No intermediate form has ever been found between scales and hairs, and the proposal was only based on the development of sensory bristles in the hinge scale region of reptiles. In contrast, the elongated scales of *Longisquama* from Triassic 240 mya, compared to the normal growth of buds during feather ontogeny, suggested that the elongation of a preexisting scale could have led to feathers [66]. A correlated view is that avian scales are directly related to squamate scales (among others: [67,68,69]). For several years I defended two views that oppose the classical ones: that hairs and feathers do not derive from squamates scales, and that feathers are the point of origin for avian scales [56]. 

**Figure 3:**
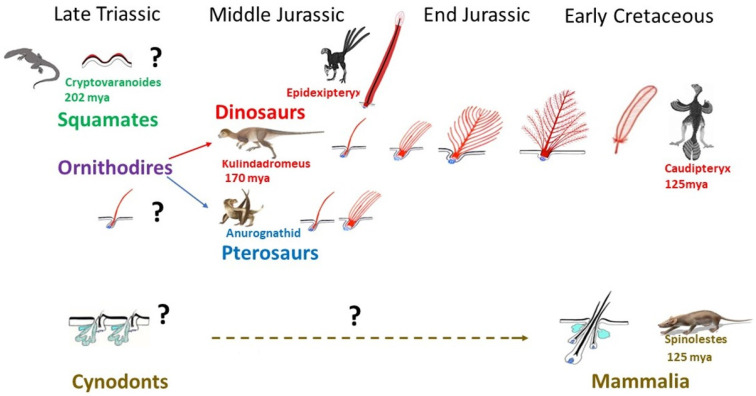
In contrast to the evolution of feather, hair and squamate scale morphogenesis appear to have not particularly varied. Squamates originate in Late Triassic, and we can presume that they were covered by epidermal scales. Fossils of pterosaurs and early dinosaurs indicate that they were fluffy animals, so we can presume that single barbs were present in their common ancestor, the ornithodires. The evolution of feather architecture appears to not follow the same path, and the rachis formation can precede or not the formation of the barbules. Moreover, several types of feathers do not exist as the ribbon feathers of Epidexipteryx anymore. In modern mammals, hair has a simple architecture which was already present in Early Cretaceous mammalia. Credits: Kulindadromeus by courtesy of P. Godefroit, Anurognathid by courtesy of M. Benton, Spinolestes, by courtesy of T. Martin. Schema of a ribbon like feather of Epidexipteryx, modified after Xu and Guo [81]. Schema Dhouailly.

Very rapidly after the first sauropsids appeared, c. a. 320 mya, they split into squamates and archosaurs. The oldest known lizard, *Cryptovaranoides,* was living in Triassic, 202 mya [70], but unfortunately this fossil, which comprises a partial head skeleton with unique squamate traits, does not concern skin. Until now, the oldest fossilized scaled integument has been only found in Late Cretaceous mosasaur *Ectenosaurus* [71]. Like those of modern squamates, mosasaur scales varied across the body in type and size. The keeled scales covering the upper regions of the body and smooth scales overlay the lower. An unusual preservation of a squamate skin which dates only about 50 mya (Eocene) shows scales, which are typical of the modern Shinisaurians group [72]. Thus, we can only presume that the appearance of scales in squamates was occurring as soon as Triassic and had not varied greatly during several mya (Figure 3). In Triassic a burst of life recovery occurred from end-Permian mass extinction, involving vertebrates such as squamates and the pterosaurs, as well as the first dinosaurs, and the survivor cynodonts which will give rise to mammals in the Jurassic. Pterosaurs have long been recognized as fluffy animals, covered with filaments previously named pycnofibers [73,74], which were recently recognized as feathers comprising isolated filaments or a bunch of simple filaments [75] (Figure 3). Among dinosaurs, the few giant sauropods for which integument is preserved have polygonal imbricated scales [76,77,78]. However, thousands of recently discovered astonishing fossils have shown that theropods (avian lineage), sauropods, and even a basal ornithischian like *Kulindadromeus* [51] had feathers (Figure 3). Did all dinosaurs have feathers? Just because we do not find feathers fossilized does not mean they were not there. They are so hard to preserve that exceptional circumstances are required for them to be found. Moreover, the presence of epidermal scales in a large adult individual does not rule out the possibility that younger individuals possessed feathers. Another possibility is that *Tyrannosaurus rex* and its closest cousins were so big that they could have lost their feathers to prevent overheating—the way that elephants have reduced their coat of hair. A 130 mya small primitive tyrannosaur, *Dilong*, was covered from head to tail with downy fluff and primitive feathers [79]. Filamentous feathers on some large tyrannosauroids from China have raised the possibility that similar integumentary structures were widespread throughout the group, even among the largest Late Cretaceous tyrannosaurids. A feather origin has thus been sought for ornithodires, the common ancestor of pterosaurs and dinosaurs, during the Early Triassic, about 250 mya [80] (Figure 3).

Protofeathers are simple filamentous structures filled with corneous beta proteins and melanosomes which can be assimilated to single barbs, about 100 µ width/15 mm long, in *Kulindadromeus* [51]. The protofeathers can thus be qualified as hair-like feathers as they appear to have no tubular structure. Several authors wrongly suggested this as being part of the scale to feather origin theory (among others: [68,82,83,84]). These single barbs eventually evolved into branched structures, until the pennaceous feather. This typical feather consists of a central shaft (rachis), with serial paired branches (barbs) forming a flattened, usually curved surface—the vane. The barbs possess further branches—the barbules—and the barbules of adjacent barbs are attached to one another by hooks, stiffening the vane and forming the most complex cutaneous appendage yet to be produced during evolution. It should be noted that the evolution of the feather architecture appears to not always follow the same path, and the rachis formation can precede the formation of the barbules. Moreover, some dinosaurs had feathers that are not seen in modern birds, with the best example being the ribbon-like tail feathers of a maniraptora, *Epidexipteryx* from Middle to Late Jurassic [85]. Theropods show a great diversity of feather types [86], and while the clade *Coelurosauria* shows the same simple feather types as ornithischian dinosaurs and pterosaurs, some show pennaceous feathers, as seen in modern birds. Their functions are varied and correspond to different types of feathers (Figure 3). The first to have appeared during evolution, in ornithodires, was insulation with plenty of single barbs, then with down feathers, which display barbules but lack hooks. Flight was the last to appear in theropods, with the formation of hooks which stiffen the vane in remiges (wing feathers), forming a strong surface for air and rectrices (tail feathers) for control of flight direction. In addition, several other functions involve sensation (bristles), sexual attraction, and camouflage (colored feathers). Moreover, erectable feathers form fans that are linked to sexual courtship or fight.

Both paleontological [87] and genetic evidence [88], as well as developmental biology experiments [55], show that avian overlapping scales, made of corneous beta proteins, are secondarily derived from feathers [56] rather than vice versa. Thus, during the evolution of Avialae, leg feathers were reduced in a proximal to distal direction, with appearance of overlapping scales [87]. Several different types of laboratory experiments easily led to the formation of feathered scales. Retinoic acid treatment in chick embryos at the time of appearance of leg overlapping scales increases the expression of *Shh* [55] and leads to the formation of one to three feathers, or sometimes more fused feathers, growing on the scale tip [53,55]. Ectopic expression of *Wnt/beta-catenin* [89], *Notch/Delta* pathway activation [90], or *BMP* pathway suppression [91] can convert avian scales to feathers. It should be noted that the conversion of a whole scale into one feather was never observed. Moreover, the reverse conversion of feathers into scales was never obtained. At the extreme, in culture toxic conditions of embryonic skin, several feather buds can fuse, forming oblong structures like scales, but forming feather-type corneous beta proteins [92]. Ectopic *Spry2* and *β-catenin* infection can induce new outgrowth not only from chicken scales but also from alligator scales [93]. However, in this context, while chicken scales form barb ridges, the alligator skin just forms elongated scales.

As was the case of feathers preceding birds, hair preceded mammals (Figure 3). However, until now, only a few specimens of the mammal lineage have been discovered with fossilized fur. After the proposal of the origin of hair based on the formation of sensory bristles in the scale hinge region [65], hair was suggested to arise from reptilian claws [94]. This was based on the finding of alpha hair-like proteins in these mostly beta-keratinized structures. However, these data do not prove that an evolutionary link between hair and reptilian claw exists. Cysteine-rich alpha-keratins are not restricted to mammals, meaning that the evolution of hair involved the co-option of pre-existing proteins, which might have been present in a basal amniote, i.e., a common ancestor of synapsids and sauropsids. Less classically, hairs have been proposed to originate from the innervated conical keratinized structures of basal amphibians [95], or from a component of a sebaceous gland apparatus [96]. These two last propositions appear better founded. Indeed, hair follicles cannot be dissociated from sebaceous gland because they display an integrated working mechanism. In living mice, a single placode gives rise to hair follicle and its associated sebaceous glands [97], and all mouse lines with sebaceous deficiency present progressive scarring alopecia [17,18,98]. This integrated development indicates an ancient association, but not a gland-to-hair evolution as previously postulated [96]. Primitive hairs might have developed in conjunction with skin glands in basal synapsids. A Mammaliaform, *Castorocauda* of Middle Jurassic 160 mya [99], was discovered surrounded by a dense fur *halo*, and the first function for hair was insulation. The Early Cretaceous *Spinolestes* belonging to Mammalia 125 mya showed not only dense fur, but remarkably intact guard (primary) hairs and secondary hairs with their bulbs (Figure 3). Their shafts presented different cuticle patterns [10]. This fossil also presents oval horny “scales”, protospines and hair associated to skin folds, i.e., a polymorphism proper to some extant mammals and associated to protection against predators. Hair appears to be at the origin of “scales”, spines, or horn in various modern mammal species. Thus, in contrast to the evolution of feather, from a simple filament to progressive degrees of branched structures, and even to different shapes which do not exist in modern birds, the hair shaft, a simple filament, covered with cuticle cells appears to have not varied, at least from Early Cretaceous. Only the discovery of well-preserved fossils belonging to the sequential radiations of synapsids—pelycosaurs, therapsids, and cynodonts—will confirm if primary hair might have consisted of a corneous wick at ductal openings of glands, functioning to allow the sebum flow. The only known evolutionary modification of hair follicle/gland complex gave rise to the mammary gland [100]. The independence of mammary glands versus hair follicles was acquired only during Cretaceous with the appearance of eutherians. In modern monotremes the mammary gland corresponds to a simple ventral patch of hairy skin producing milk. In marsupials the mammary glands are individualized, but still coexist in young specimens with hair follicles [101].

## 4. Squamate, Avian, and Mammalian Ectoderms Are Genetically Programmed to Build Scales, Feathers, or Hairs, Respectively, and Their Early Morphogenesis Pathways Were Conserved during Evolution

The first cell biology research about the morphogenesis of cutaneous appendages in the seventies was related to cell interactions between the two skin components—the dermis, and the epidermis. Analyzing the results of heterospecfic dermal/epidermal recombinations between chick, mouse, and lizard (*Lacerta muralis*) embryos, I was the first to pinpoint [102,103,104] that: (1) the clade type of cutaneous appendages is determined by the epidermis which presents a default competence; (2) the cutaneous appendage morphogenesis requires a continuous dialogue with the dermis, organized in two major steps; and (3) the first step of this dialogue has been conserved during evolution. Later, molecular biology studies have shown the developmental pathways confirming this reciprocal transfer of messages. These cascades principally include Wnt/β-catenin, Ectodysplasin (Eda/Edar), Fibroblast growth factor (FGF), and Shh signaling (see Section 5). 

**Figure 4:**
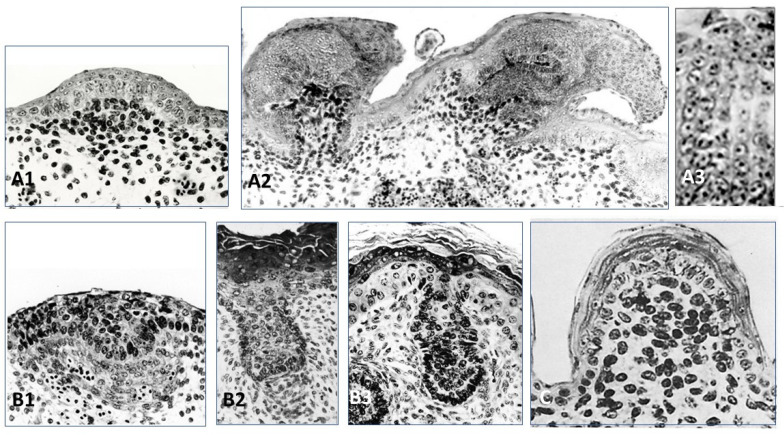
Two major steps in feather and hair morphogenesis, but only one in squamate scale. Xenoplastic dermal epidermal recombinants cultured on chick chorioallantoïc membrane. Note the darkly staining of mouse nuclei. (**A1**) E7 chick epidermis recombined with E14.5 mouse dermis leads after 4 days of culture to the formation of feather placodes associated to mouse dermal condensation. (**A2**) After 8 days of culture, abnormal short feathers are observed with recognizable alignment of cells (**A3**), characteristic of barb ridges. (**B1**) Recombinant of E12 mouse epidermis and E7 chick dermis leads after 3 days to formation of hair placodes associated to chick dermal condensation then (**B2**) to hair buds still associated to a chick dermal condensation. (**B3**) However, after 8 days of culture the dermal papilla has been dispersed, and despite an elongation of the hair bud, the hair morphogenesis was interrupted. (**C**) Recombinant of E 17 lizard epidermis and E14.5 mouse dermis leads, after 8 days, to the formation of scales with their corneous layer. **Credits**: After Dhouailly D, 1973, [102] and Dhouailly D, 1975, [103].

The recombinant of E10 umbilical cord chick epithelium with a E12.5 mouse dorsal dermis does not form any appendages, while its association to a E14.5 mouse dorsal dermis formed short abnormal feathers [102]. Thus (1) the ectoderm from the chick umbilical cord might be deprived of Wnt/β-catenin signaling, and (2) at E14.5 the dorsal mouse dermis is already educated by its own epidermis to form dermal condensations [105] and does not need any more this ectodermal signaling. Recombinants of E6 chick dorsal epidermis and E12.5 or E14.5 mouse dorsal dermis after 4–5 days of culture lead to the formation of feather placodes associated with mouse dermal condensations [102] (Figure 4A1). After eight days of culture, abnormal short feathers were observed (Figure 4A2). They involved a few chaotic barb ridges, recognizable by their characteristic alignment of cells (Figure 4A3). The control of barb ridges morphogenesis depends on dermis, as shown by the results of dermal/epidermal recombinants between chick and duck embryos. In chick and duck, the neonatal feather is downy, but is radially symmetric, or bilaterally asymmetric, possesses a rachis (or not), and presents ten or twenty barbs, respectively. All these structures are governed by the dermis [106,107]. Results from experiments using adult chick and duck forming follicles during regeneration after plucking are consistent [108] with the results of embryonic chick/duck recombination. A *Wnt3a* expression gradient is higher in the anterior rachis side and is in accordance with the duck origin of the dermal papilla. 

The overexpression of its antagonist *Dkk1* results in a gradual conversion from the bilateral symmetric feather to radially symmetric feather [108]. The formation of barb ridges involves Shh and BMP2 signaling, with *Shh* being expressed broadly in the entire marginal plate epithelium, while *Bmp2* is expressed exclusively at the peripheral bend of the marginal plate epithelium, where it overlaps with *Shh* expression [67]. These two signaling pathways act according to an activator-inhibitor model—Shh is the activator, which upregulates its own transcription and that of *BMP2*, whereas BMP2 down-regulates *Shh* expression [109]. It should be noted that the making of a pennaceous feather, with its increasing branching complexity, barbs, barbules, and barbicels (hooks), requires multi-level specifications, involving Noggin, BMP, Wnt3a, Wnt2b, and finally Gremlin, which were successively established during evolution [110]. Interestingly, we demonstrated an autonomous transformation of the chick amnion into a typical skin with downy feathers [111]. Such a metaplasia requires the same molecular influences: Noggin is needed to counter the BMP4 pathway in the amniotic mesoderm, and Shh is responsible for stimulating the proliferation of cells. Finally, the activation of the ectoderm and mesoderm cells of the amnion leads to the formation of the most complicated neonatal skin appendages: downy feathers. We never observed a rudimentary scale morphogenesis.

Recombinants of E12.5 mouse epidermis and E7 chick dermis [102] led to the formation of hair placodes after 3 days (Figure 4B1), then to hair buds still associated to a chick dermal condensation (Figure 4B2). However, after 8 days of culture the hair bud is longer than usual, but the dermal papilla is dispersed, and the hair bud morphogenesis is interrupted (Figure 4B3), contrary to controls which differentiate hair follicles. In normal development, the extremity of the hair bud surrounds the mesenchymal dermal papilla, a key signaling and organizing center to form the hair bulb [112,113]. Within the bulb, the hair matrix progenitor cells, which receives signals from the dermal papilla, will terminally differentiate into cells that form seven concentric layers, first into the companion layer, and then into the hair shaft and the inner root sheath [114]. How these choices are made is not entirely elucidated. It has been suggested that the location of matrix progenitors along the hair follicle medial-lateral axis largely governs their fate [115], and that signaling gradients may lead to differential expression of matrix transcription factors such as Msx2, Dlx3, Wnt5a, and Foxn1 [116,117,118,119]. 

The recombination of E17 lizard (*Lacerta muralis*) embryo epidermis and mouse or chick dermis leads to the formation of scales, which after 8 days are cornified [103]. Their shape is round, with a mouse (Figure 4C) or chick dorsal dermis, or rectangular with a chick tarsometatarsal dermis. More than fifty reverse recombinants of lizard dermis from E15 to E26 (i.e., from flat to “undulated” skin, i.e., contiguous bumps) with E6–E7 chick dorsal epidermis or 12.5–13.5 mouse dorsal epidermis were analyzed. They led mostly with the E15–E20 lizard dermis to a few very abnormal in-growth of the chick epidermis, and to a few hair-like buds of the mouse epidermis. An explanation could be the absence in lizards of mesenchymal condensations associated with the placodes. Until now, epidermal signaling expressions, such as Shh, Ctnnb1, and Eda/Edar, have been shown in squamate scales, but dermal BMP4 signaling under the placode needs to be confirmed [120]. Squamate scales, as is the case for other short appendages, avian reticula [54,55,121], and mammalian fingerprints [122], are not associated with a dermal condensate, could mainly be formed by intra-epidermal signaling, involving all the classical placodal pathways. Finally, as feather and hair are the default competences of avian and mammalian ectoderm, scale is the default competence of squamate ectoderm. 

## 5. Placode Formation, a Dynamic Process Common to All Vertebrate Cutaneous Appendages

Before placodes formation, the dermal cells colonize the space below the ectoderm, following a migration from various origins according to the different body parts. This early morphogenesis of the integument has been extensively studied in chicks and mice. The zones of origin are the dermomyotome for the back for chick [123], and for mouse [124], the somatopleural mesoderm for the ventral trunk [111], the neural crest for the head, including buccal region and cornea (among others: [125,126]). In the chick dorsal region, the dermis originates from different regions of the dermomyotome between E3 and E5, in response to dorsal Wnt signals from the neural tube and the ectoderm (among others: [127]). A densification of the dermal fibroblasts occurs by E6 throughout the area of the future dorsal feather field. Dense dermis formation and maturation are concomitant with expression of the twist-like bHLH transcription factor *Dermo-1* in the sub-ectodermal mesenchyme, not only in mouse and chick [128,129], but as recently shown in Zebrafish [130]. A low-cell density dorsal dermis as in the chick Ottawa naked mutant is unable to support feather development when associated to a wild-type epidermis [131]. A dense dermis formation never occurs in the chick midventral apterium that results from ventral closure of the body and considered as a scar [132] but occurs in the skin of Scaleless chick embryo [133].

**Figure 5:**
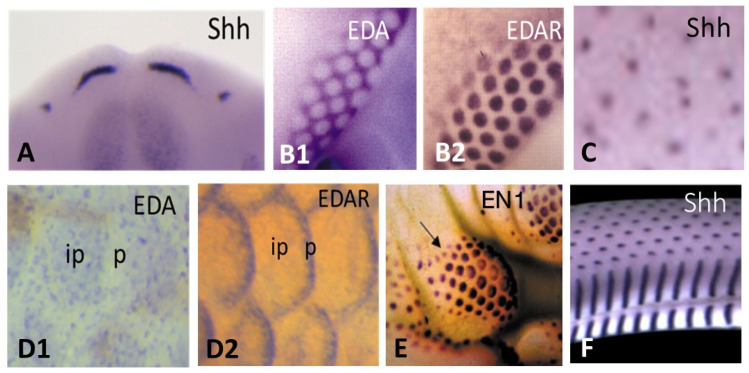
Different placodal shapes revealed by the expression of markers in different vertebrates. (**A**) Oval dental placodes of incisors of E12 mouse, as revealed by *Shh* expression. (**B1**,**B2**) Round femoral feather placodes of E7.5 chick as revealed by EDA in the inter-placodal and EDAR in the placodes. (**C**) Round dorsal hair placodes of E14.5 mouse, as revealed by *Shh* expression. (**D1**,**D2**) Crescent placodes of a 9–10 mm fry zebrafish, as revealed by the expression of *EDA* in the inter-placodal (ip) and of *EDAR* in the placodes (p). (**E**) Round placodes of plantar reticula of a E14 chick are revealed by the expression of EN1. The arrow indicates the central foot pad. (**F**) Round dorsal and rectangular ventral placodes of a E26 snake are revealed by *Shh* expression. **Credits**: (**A**) by Courtesy of Dr. I. Thesleff; (**B1**,**B2**) After Houghton et al., 2005, [134], reprinted with permission of the company of biologists; (**C**) After Huang et al., 2012 [135]; (**D1**,**D2**) by courtesy of Dr. M. Harris, Harris et al., 2008 [64]. (**E**) After. Prin and Dhouailly, 2004 [55]. (**F**) from the work of Di-Po and Milikovitch, 2016 [120].

The first stage of cutaneous appendages formation in all vertebrates is an epithelial placode. The shape of the placode varies—oval for mouse tooth incisor (Figure 5A), and mammary gland [49], round for feather (Figure 5B1,B2) (among others: [134]), and hair (Figure 5C) (among others: [135]), rectangular for avian overlapping scales [136]. Anatomically, the teleostean placode, avian reticula and squamate placodes are not distinguishable, but are revealed by the expression of conserved pathway markers, illustrating an oval evolving in crescent shape in zebrafish (Figure 5D1,D2) [19,64], a round shape in chick reticula (Figure 5E) [54,55,137], a round dorsal and rectangular ventral in squamates (Figure 5F) [120]. Likewise, placode thickness varies from one layer of elongated cells that form arcades in feather [138], one layer of slightly elongated cells in avian epidermal scale [136], and about three cell layers in hair (among others: [139]). It should be noted that despite apparent morphological discrepancies the placode can be detected by the expression of identical molecular markers. The dermal cells underlying the placode form a condensation in odontode [140], tooth (reviewed in [40]), hair (among others: [139]), or feather [138]. A slight dermal condensation was observed in teleostean scale [19,64], avian overlapping scale [136], and mammary glands [141]. It should be noted that for incisor teeth and hair follicles, a strong *activin* expression was shown in the dermal papilla [142,143] and appears linked to a sustained high activation of *Shh* in the epithelial component. In contrast, no dermal condensation forms in non-protruding cutaneous appendages appeared to be linked to a low level of epidermal *Shh* activation, at least in avian plantar reticula [54,55,137] and mammalian fingerprints [122].

In chick embryos, of the two primordia components, the placodes are larger and earlier than the dermal condensations. In the initial middorsal row at E7, one to three placodes precede the appearance of dermal condensations, and as the lateral rows successively differentiate at E7.5, one row of epidermal placodes precedes each row of dermal condensations [144]. The placode results in the chick embryo from an elongation of the apico-basal axis of the epidermal cells, due to a mechanical compression, which causes a rapid displacement of β-catenin protein from cell junctions to the nuclei [145]. In the mouse, the hair placode has been shown to be initiated by the exit of the epithelial cells from the cell cycle. Its formation is then driven by cell migration and not by cell proliferation [146]. For a long time [147,148], it has been known that no cell proliferation is involved in chick or mouse dermal condensation formation, but only by redistribution of dermal fibroblasts. The motility of fibroblasts is regulated by the expression of *FGF20* by the epidermis [149]. By contrast, in shark odontodes, the primordia formation results from localized epithelial and mesenchymal cell proliferation [61]. 

A great number of pathways have been shown to intervene in the development of cutaneous appendages. Among them, the Wnt/β-catenin signaling appears to be fundamental, first in the ectoderm, then in its dense mesenchyme to allow the formation of all types of cutaneous appendages in all species that have been studied (among others: [21,41,150,151,152,153]). Using the *dkk1* transgenic line that allows for conditional expression of a potent and selective Wnt/β-catenin signaling inhibitor [154], it was shown that inhibition of Wnt/β-catenin signaling during cutaneous appendage development delays feather regeneration [155] and prevents teleostean scale and hair formation [21,35]. Reversely, the forced expression of *Wnt/β-catenin* in mouse or chick epidermis leads to the formation of new hair or feather follicles, respectively [89,156].

**Figure 6:**
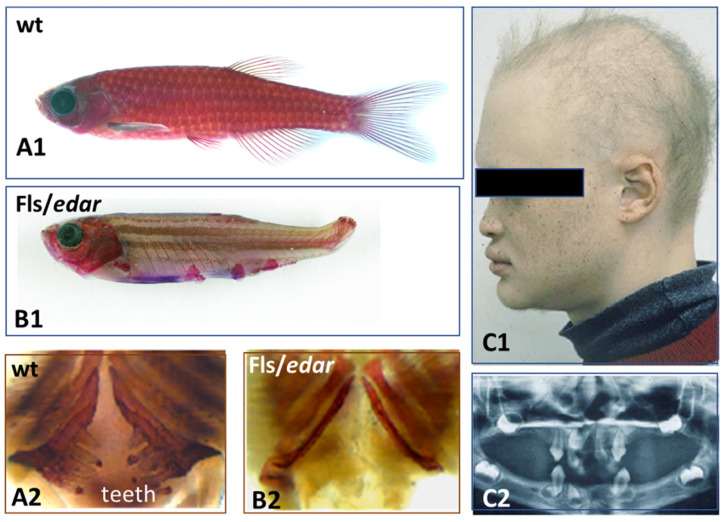
When EDA or EDAR are missing, the results are identical in Zebrafish and in Humans. Cutaneous appendages morphogenesis is deficient in absence of EDA/EDAR signaling. Rare dermal scales in Zebrafish (compare figures **A1**, **B1**), with a total absence of teeth (compare figures **A2**, **B2**). Rare hair (**C1**) and teeth (**C2**) in Human. Credit: by courtesy of M. Harris, 2008 [64].

Afterwards, the placode formation involves, principally and successively, the Eda/Edar, FGF20, and Shh signaling, as recently emphasized both in chick feather formation [145] and zebrafish scale formation [21]. Within this sequence, the Eda/Edar signaling pathway is presented as playing a pivotal role. Indeed, the mutation of *Eda* or *Edar* results in zebrafish [64], bearded dragon [120], and humans [157] having a deficiency of teeth and body appendages (Figure 6), respectively dermal scales, epidermal scales, hair and sweat glands. 

**Figure 7:**
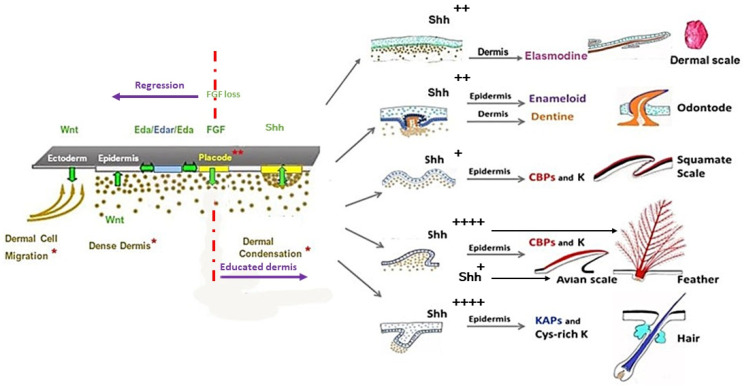
The first group of developmental pathways, leading to cutaneous appendage buds, is common to all vertebrates. The absence of Wingless-integrated (Wnt) prevents dermis formation and placode initiation in all types of appendages in all species. The EDA/EDAR pathway, activated downstream of Wing signaling, triggers several pathways, among others FGF and Shh, which are required both for the formation of the dermal condensation and the growth of the placode (**), respectively. FGF plays a pivotal role (see text). A second group of pathways governing the specific architecture of each kind of cutaneous appendage varies, not in type, but in location and timing, mostly Wnts, Shh, BMPs, Noggin, Gremlin. The regulation of *Shh* is of critical importance for the growth of the different appendages, from low (+), to medium (++), to very high (++++). Note that the simple architecture of squamate epidermal scales does not involve an elaborate second group of messages. * Three steps of dermis formation. Note that dermal condensation does not exist in squamates. CBP: corneous beta proteins; K: keratins, KAPs: keratin associated proteins, cys-rich K: cysteine rich keratins. Schema: Dhouailly.

However, I suggest that the pivotal role is better played by FGF 20 signaling for two major reasons. First, it has been shown that the signaling cascade goes backwards in the absence of FGF 20. The chick Scaleless mutant lacks feathers and foot scales because of a developmental failure of epidermal placode formation (among others: [158,159]) due to a loss-of-function mutation in *FGF20* [160]. In this mutant, the *Eda/Edar* signaling is expressed in time, but rapidly disappears [161]. Second, after receiving the FGF20 signaling [149] the chick or the mouse dermis is educated and memorizes its aggregation ability and its role in cutaneous appendage formation for several days, as shown in my group [33,103,131,162]. In recombination experiments using an E14.5 mouse dorsal dermis, the automatic dispersion of dermal condensations after skin dissection leads to their reorganization. This process takes a few hours when the dermis is recombined to a chick embryo epidermis, even from the umbilical region [102], and 8 to even 12 days when recombined to an adult rabbit corneal epithelium [33], inducing, respectively, the formation of short abnormal feathers and perfect hair follicles. Thus, after receiving the FGF20 signaling, the dermal fibroblasts can keep in memory at least during several days the ability to form condensations: they are “educated” (Figure 7).

Placode formation appears in a pattern that is first determined in the epidermis and is then transmitted to the dermis. This was shown particularly well in the recombinants of lizard epidermis either with a E14.5 dorsal mouse dermis, a E12.5 upper lip mouse dermis, or a E7.5 dorsal chick dermis, thus with an “educated dermis”. In those cases, the recombinants exceeded the stage of the acquisition of the dermal patterning and lead to the formation of scales distributed according to patterns of mouse pelage hair, of mouse vibrissae, or of dorsal feather, respectively [103]. In the chick, most feathers are restricted to specific area of the skin, the feather tracts, or pterylae. For years, the beautiful hexagonal pattern of placodes in the chick spinal pteryla [138] had been the object of numerous works. The spinal pteryla is surrounded by semi-apteria, whereas the ventral skin has a true medial apterium framed by the ventral pterylae. Both dorsal and ventral pterylae appear in successive rows of placodes, parallel to the body sagittal plane. A different pattern is observed in the central foot pad of the chick embryo, where a first central placode radiates outwards [53,54,55,137]. A supplementary pteryla forming successive concentric rows of placodes can be initiated by the graft of Noggin engineered cells in the midventral apterium of the chick embryo [111]. The organization in placodes is a dynamic skin self-organizing process. Its loss after dissection of the embryonic skin and its re-initiation in vitro shows that this process is autonomous [131,162].

Twenty years ago, Dr. Chuong and his colleagues started to classify different signaling pathways as activators or inhibitors during feather initiation [163]. The FGF20 pathway acts as an activator, and its epidermal expression promotes dermal condensation formation via its chemoattractant effect on fibroblasts [133,164]. The BMPs have generally been considered as inhibitors of feathers [163], as well as of hair [165] and tooth formation [166]. A local high concentration of BMP4 with a coated bead led to the formation of a glabrous area [163]. Reversely, an ectopic feather-forming dermis can be obtained by the inhibition of BMP4 signaling with Noggin engineered cells in the mid-ventral apterium or with Noggin and Shh engineered cells in the amnion [111]. The patterning of cutaneous appendages is thought to be controlled by the operation of Turing, or reaction-diffusion system, based on interactions between differentially diffusing activator and inhibitory morphogens, giving rise to autonomous pattern formation, evolving from chondrichthyans to mammals. Among others, we have proposed a reaction-diffusion system, which not only simulates feather patterning, but which also can account for the negative effects of excess BMP2 or BMP7 on feather formation [167]. We have shown that BMP7 and BMP2 play antagonistic roles during feather primordium formation. BMP7 appears to act as a chemoattractant factor for dermal fibroblasts, while the expression of BMP2 in dermal condensations triggers the arrest of dermal cell migration [167]. This is consistent with their timing of expression: *BMP7* dermal expression is regulated by canonical Wnt signaling derived from the placode and appears earlier than *BMP2*. An elegant recent work of Dr. Headon and his colleagues uncovers a process in which elements of both reaction-diffusion and cell movement-based patterning systems are integrated into a unified periodicity-generating mechanism [145]. In the dorsal skin of chick embryos, a travelling wave of *EDA* expression on each side of the midline interacts with a receding wave of *β-catenin/EDAR* to trigger the *FGF20* epidermal expression, thus shaping a regular hexagonal pattern of placodes formation. The same integrated system applies for duck, but not in ostrich and emu, which display a much less regular pattern [145]. The EDA role is also distinct in mice, as its activity has been shown to be required for hair placodes development but not for patterning [168]. Interestingly, pelage guard hair patterning was shown to result from the interplay between a reaction–diffusion system in the epidermis and a mesenchymal self-organization process, driven by a network of FGF, WNT, and BMP interactions [168]. The process of scale patterning in squamates remains to be studied, and is likely to result only from intraepidermal signaling, as shown in the formation of fingerprints in mammals [122]. Finally, there is not only a great diversity of vertebrate appendage patterns in each species and each different skin regions, but also variations in the patterning processes.

## 6. Conclusions

The epidermal placode formation is a dynamic and instable process, common to all vertebrate cutaneous appendages. The conservation of a cascade of signaling pathways underlying the first stage of teeth and all skin appendages across phylogenetically distinct vertebrates, from chondrichthyans to mammals (Figure 7), indicates its origin in a common ancestor. Once the spatial pattern of placodes has been defined, the selected cells activate expression of other genes, both in the placode itself and its associated mesenchyme, promotes their development and constructs the mature cutaneous appendage. Later, a divergence in developmental processes gave rise to a plethora of diverse skin appendages. Such a divergence is due to the different level, timing, and location of expression of the same actors, among them principally Shh, BMPs, Dkks, Wnts, and Notch. A sustained expression of the Shh pathway in the placode, activated by factors issued from its associated dermal condensation, both in birds and mammals, results in the formation of long, protruding appendages. This explains the formation of protruding short feather filaments, and of elongated hair buds in xenoplastic dermal/epidermal recombinants between chick and mouse. The chick epidermis started to form barb ridges as it was genetically programmed. However, as it lacked adequate dermal signaling, among others Wnt3a/Dkk1, the barb ridges were abnormal and chaotic, and the feather morphogenesis stopped. Likewise, the elongated hair bud loses its interaction with the dispersing chick dermal condensation, in contrast with the engulfing of the dermal papilla that allows the normal pursuit of hair follicle morphogenesis. It should be noted that all the non-protruding appendages develop, without an association to a mesenchyme condensate, as squamate scales. This can explain the inability of the lizard dermis to interact with a chick or mouse epidermis, in contrast with the reverse recombinants. Finally, several experiments have shown that the ectoderm displays a default competence to form epidermal scales in squamates, feathers in birds, and hair in mammals. A relevant question is how this default competence is genetically programmed, and how to interpret the persistence of a tooth potential in oral ectoderm. Perhaps the answer will come from the comparison of teeth and odontodes potentials in oral versus body ectoderm in sharks.
